# *Tumidusternus*, a new genus of Aspidimerini from China (Coleoptera, Coccinellidae)

**DOI:** 10.3897/zookeys.511.9582

**Published:** 2015-07-02

**Authors:** Lizhi Huo, Wenjing Li, Xiaosheng Chen, Xingmin Wang, Shunxiang Ren

**Affiliations:** 1 Engineering Research Center of Biological Control, Ministry of Education; College of Natural Resources and Environment, South China Agricultural University, Guangzhou 510642, China

**Keywords:** Coleoptera, Coccinellidae, Aspidimerini, *Tumidusternus*, new genus, new species, China

## Abstract

*Tumidusternus* **gen. n.**, along with *Tumidusternus fujianensis* **sp. n.** (Coleoptera, Coccinellidae, Aspidimerini) from China is described and illustrated. A key to the tribe Aspidimerini is given.

## Introduction

Aspidimerini was erected by Weise (1900) with two genera: *Aspidimerus* Mulsant, 1850 and *Cryptogonus* Mulsant, 1850. Kapur (1948) revised this tribe and erected two new genera: *Pseudaspidimerus* and *Acarinus*. Huo et al. (2015) added another genus *Trigonocarinatus*. This tribe can be characterized by: (1) legs with trochanters and femora extremely broad and flattened, forming together subrectangular or oval clubs; tibiae and tarsi can be completely hidden under the trochanter-femur club for protection; tibiae flattened, tarsi 3-segmented, tarsal claws bifid; (2) Antenna very short, 8 or 9 segmented, hidden in antennal grooves, partially visible in ventral view; scape large, transversely oval, pedicel smaller and subtriangular, antennal club fusiform or clavated; (3) Abdomen with 6 ventrites in both sexes; ventrite 1 distinctly longer than ventrite 2, with hind margin arcuate posteriorly.

During our recent study on Aspidimerini, an unusual species was discovered, which prosternum is extremely tumid, highly raised above the ventral surface. A further comparison of more characters (e.g. prosternum, mentum, antenna and legs) with other genera of Aspidimerini revealed that this species is distinctive. Hence, a new genus, *Tumidusternus* gen. n., is here proposed to accommodate this unusual species.

## Material and methods

All studied materials were collected from China. Type specimens designated in the present paper are deposited in the Department of Entomology, South China Agriculture University, Guangzhou and the Institute of Zoology (IOZ), Chinese Academy of Science, Beijing.

External morphology was observed with a stereomicroscope (SteREO Discovery V20, Zeiss). Measurements were made using an ocular micrometer attached to the stereomicroscope as follows: (TL) total length, from apical margin of clypeus to apex of elytra; (TW) total width, across both elytra at widest part; (TH) total height, through the highest point of elytra to metaventrite; (HW) head width, including eyes; (PL) pronotal length, from the middle of anterior margin to the base of pronotum; (PW) pronotal width at widest part; (EL) elytral length, along the suture, from the apex to the base including the scutellum; (EW) elytral width, across both elytra at widest part; (ID) interocular distance, nearest distance between two eyes. Male and female genitalia were dissected, cleared in 10% solution of NaOH by boiling for several minutes, and examined with an Olympus BX51 compound microscope. Images were photographed with digital cameras (AxioCam HRc and Coolsnap-Procf & CRI Micro*Color). The software AxioVision Rel. 4.8 and Image-Pro Plus 5.1 were used to capture images from both cameras, images were cleaned up and laid out in plates with Adobe Photoshop CS5. Morphological terms follow Ślipiński (2007) and Ślipiński and Tomaszewska (2010).

## Taxonomy

### Tumidusternus

Taxon classificationAnimaliaColeopteraCoccinellidae

Huo & Ren gen. n.

http://zoobank.org/6F12A466-6CCD-43D2-8691-658D1B3E2833

Figures 1 , 2

#### Type species.

*Tumidusternus fujianensis* Huo & Ren, sp. n.

#### Diagnosis.

**Figure 1.**
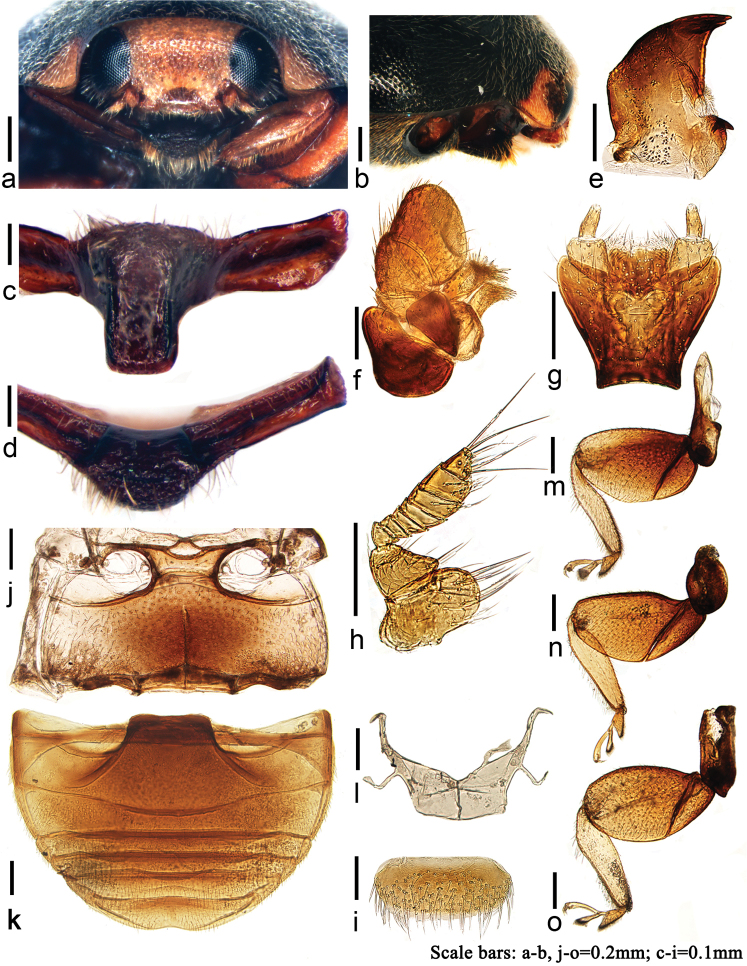
*Tumidusternus fujianensis* Huo & Ren, sp. n. **a** frontal view **b** anterior half of body, lateral view **c** prosternum **d** prosternum, anterior view **e** mandible **f** maxilla **g** labium **h** antenna **i** labrum **j** mesoventrite and metaventrite **k** abdomen, male **l** metendosternite **m** front leg **n** mid leg **o** hind leg.

*Tumidusternus* can be easily distinguished from other genera of Aspidimerini by middle part of prosternum extremely tumid, highly raised above the ventral surface (Fig. 1a–d) and anterior margin of mentum triangularly emarginate (Fig. 1g). While in *Acarinus* and *Cryptogonus*, middle part of prosternum is flat and anterior margin of mentum has a small, rectangular notch at middle; in *Aspidimerus*, middle part of prosternum is evenly convex, but not tumid and anterior margin of mentum is truncate, without a notch or an emargination; in *Pseudaspidimerus*, only area of the prosternum between the parallel carinae lies at a higher level than the lateral parts outside the carinae, and anterior margin of mentum possesses a small, triangular emargination at middle.

#### Description.

Body small (TL: 2.73–2.95 mm), oblong oval (TL/TW=1.25–1.29) and moderately convex, dorsum finely punctate and pubescent.

**Head**. Head transverse, brown. Eyes large, rounded and finely faceted, with sparse interfacetal setae. Clypeus widely emarginate, partially covering labrum. Antenna (Fig. 1h) very short, 9-segmented, with sparse long setae at inner side, hidden in antennal grooves, invisible from above and partially visible from below. Scape large, transversely oval, 1.5 times as wide as long, pedicel smaller and subtriangular, Antennal club fusiform, distinctly longer than width of scape, terminal antennomere acutely conical, as long as penultimate one. Maxilla (Fig. 1f) with cardo and stipes subtriangular. Maxillary palp 3-segmented, always hidden under the cardo and stipes for protection, terminal palpomere securiform. Mentum (Fig. 1g) subtrapezoidal, with anterior margin widely triangularly emarginate and posterior margin slightly incurved, partially covering labium. Labial palp (Fig. 1g) 2-segmented, basal palpomere gradually thicker to apex, apex 2 times as wide as base of terminal palpomere. Terminal palpomere cylindrical, slightly tapering apically, rounded at apex. Mandible (Fig. 1e) broad with apex bifid and basal tooth pointed. Labrum transverse (Fig. 1i), 2.0–2.5 times as long as wide, covered with long and sparse setae.

**Prothorax.** Prothorax convex and transverse, anterior margin deeply emarginate, lateral margins arcuate with anterior corners rounded and posterior corners nearly orthogonal. Prosternum T-shaped with middle part extremely tumid, highly raised above ventral surface of the body, with surface coarsely punctate and densely pubescent (Fig. 1a–d). Each side folded down constituting a prosternal fold (Fig. 1c). Anterior margin of prosternum with broad border well visible in front view (Fig. 1d). Procoxal cavity distinctly transverse, longitudinal diameter shorter than prosternum in front of coxae. Prosternal process broad, width equal to length of prosternum in front of coxae, with apex rounded. Carinae parallel along 3/4 length of prosternal process then confluent with the tumid part of prosternum (Fig. 1c).

**Figure 2.**
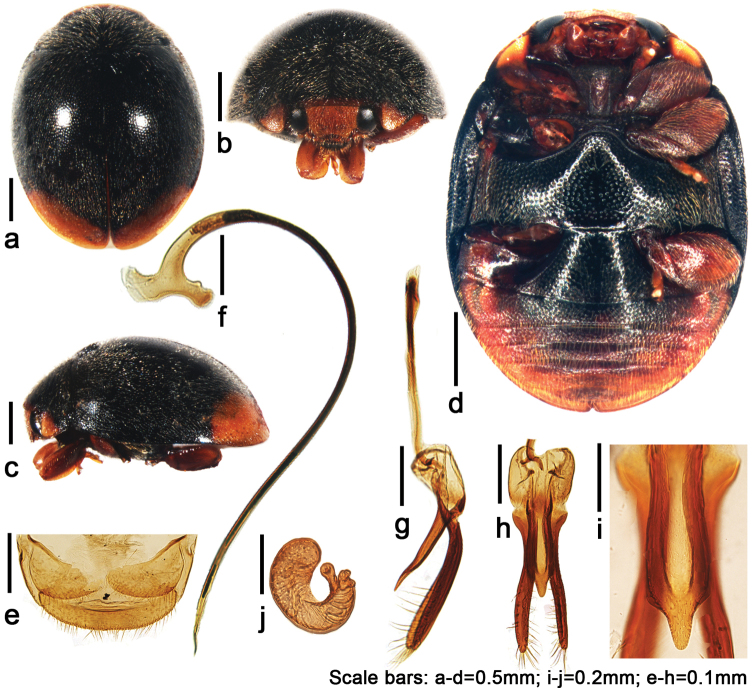
*Tumidusternus fujianensis* Huo & Ren, sp. n. **a** dorsal view **b** anterior view **c** lateral view **d** ventral view **e** ovipositor **f** penis **g** lateral view of tegmen **h** ventral view of tegmen **i** ventral view of penis guide **j** spermatheca.

**Prerothorax.** Mesoventrite (Fig. 1j) with mesoventral process 0.5 times as long as longitudinal mesocoxal diameter; anterior margin widely emarginate and concave at middle to receive prosternal process; mesoventral process as broad as mesocoxal diameter; meso-metaventral junction slightly arcuate anteriorly. Metaventral postcoxal lines joined medially, recurved and complete laterally. Discrimen long but incomplete. Metendosternite stalk 0.5 times as long as broad, tendons separated by less than width of stalk and situated on laminae (Fig. 1l). Scutellum small, subtriangular, black. Elytra moderately convex. Humeral calli weakly visible. Elytral epipleuron incomplete, gradually narrowing from base to 3/5 of elytral length, with clearly delimited cavities to accommodate apices of mid and hind femora (Fig. 2d). Wings well developed. Legs with trochanters and femora extremely broad and flattened, forming together trochanter-femur clubs. Front leg with trochanter-femur club very broad, inner margin partially straight (Fig. 1m); mid and hind leg with trochanter-femur club oval, inner margin arcuate (Fig. 1n–o). Tibia and tarsus can be completely hidden under the trochanter-femur club for protection. Tibia flattened, outer margins with groove for receiving the folded tarsus, tarsi 3-segmented, tarsal claws bifid.

**Abdomen.** Abdomen with 6 ventrites in both sexes. Ventrite 1 distinctly longer than ventrite 2, at middle 4–5 times as long as ventrite 2 and laterally slightly longer than ventrite 2. Ventrite 2 short at middle, 0.5 times length of lateral margin. Ventrites 3–4 with margins straight, equal in length. Ventrite 5–6 longer than 3–4. Ventrite 6 weakly emarginate apically in male (Fig. 1k) and rounded in female. Postcoxal lines deep but not merging with hind margin of ventrite, laterally incomplete.

#### Etymology.

The generic name is derived from Latin *tumidus* and *sternum*, referring to its tumid prosternum. Gender masculine.

### Tumidusternus fujianensis

Taxon classificationAnimaliaColeopteraCoccinellidae

Huo & Ren sp. n.

http://zoobank.org/1361BBDF-EBC3-45DD-8C06-BCD425C94C81

Figure 2

#### Types.

**Holotype**: 1♂, **China: Fujian Prov.**: Xiangxi Village, Nanjing County, [24°31.07'N, 117°17.08'E], ca255m, 18.VIII.2012, Huo LZ et al. leg.

**Paratypes** (8): 1♂2♀, with same data as holotype; **Guangdong Prov.**: 1♂, Hengshitang Town, Yingde City, [24°18.51'N, 113°22.60'E], ca185m, 5.X.2004, Ren SX et al. leg; 1♂, Shimentai Village, Yingde City, [24°25.38'N, 113°18.14'E], ca420m, 29.X.2004, Wang XM leg; 1♂ (deposited in IOZ), Guangdong Ruyuan Grand Canyon, Ruyuan County, [24°31.22'N, 113°07.38'E], ca650m, 23.IX.2005, Wang XM leg; 1♂1♀, Yinna Mountain, Meizhou City, [24°24.16'N, 116°23.40'E], ca450m, 11.X.2014, Wang XM et al. leg.

#### Diagnosis.

This species can be easily distinguished from all known Aspidimerini species by its tumid prosternum.

#### Description.

TL: 2.73–2.95 mm, TW: 2.11–2.35 mm, TH: 1.35–1.46 mm, TL/TW: 1.25–1.29; PL/PW: 0.49–0.52; EL/EW: 1.02–1.06, HW/PW: 0.59–0.62; PW/EW: 0.72–0.73. ID/HW: 0.49–0.50.

Body oblong oval, densely covered with short, silver white pubescence (Fig. 2a–c). Pronotum black with anterior margin and anterior corners yellowish brown (Fig. 2b). Scutellum black. Elytra black with apical 1/3 yellowish brown (Fig. 2a, c). Underside black except prothoracic hypomeron yellowish brown, prosternum, legs and lateral, and posterior margins of abdomen reddish brown (Fig. 2d).

Punctures on frons coarse and very dense, 0.2–0.5 diameters apart; on elytra and pronotum sparse, 1.0–3.0 diameters apart; on metaventrite densely distributed, 0.5–3.0 diameters apart. Ventral surface with short, dense, silver pubescence.

**Male genitalia.** Penis slender, curved at basal half, apex pointed (Fig. 2f). Penis capsule with inner arm slightly longer and thinner than outer one. Tegminal strut as long as main part of the tegmen. Parameres 2 times of phallobase length and 1.5 times of penis guide length, apical half with long sparse setae (Fig. 2g–h). Penis guide 4 times as long as wide, parallel for basal 3/4, then convergent to rounded apex (Fig. 2i).

**Female genitalia.** Coxites subtriangular (Fig. 2e), with dense, short terminal setae. Spermatheca curved, C-shaped, with distinct ramus and short nodulus (Fig. 2j).

#### Etymology.

The specific name refers to the holotype locality, Fujian, China.

#### Distribution.

China (Fujian, Guangdong).

#### Key to the genera of *Aspidimerini*

**Table:** 

1	Prosternal carinae absent	***Acarinus* Kapur, 1948**
–	Prosternal carinae distinct	**2**
2	Prosternal fold absent	***Trigonocarinatus* Huo & Ren, 2015**
–	Prosternal fold distinct	**3**
3	Antennal club fusiform, terminal antennomere conical, as long as penultimate antennomere; trochanter-femur club of front leg extremely broad with inner margin straight at mid length	**4**
–	Antennal club clavate, terminal antennomere rounded, distinctly shorter than penultimate one, trochanter-femur club of front leg moderately broad with inner margin curved	**5**
4	Middle part of prosternum extremely tumid, highly raised above the ventral surface of the body	***Tumidusternus* Huo & Ren, gen. n.**
–	Middle part of prosternum not tumid, only the rectangular area enclosed by carinae lies at higher level than lateral parts outside carinae	***Pseudaspidimerus* Kapur, 1948**
5	Middle part of prosternum evenly convex, carinae widely divergent anteriorly, the area between them convex and widening anteriorly to form a chin-band; trochanter-femur club with inner margin angulate at middle	***Aspidimerus* Mulsant, 1850**
–	Middle part of prosternum flat, carinae varying in outline; trochanter-femur club with inner margin perfectly arcuate	***Cryptogonus* Mulsant, 1850**

## Supplementary Material

XML Treatment for Tumidusternus

XML Treatment for Tumidusternus fujianensis

## References

[B1] Huo LZ Chen XS Li WJ Wang XM Ren SX (2015) A new genus of the tribe Aspidimerini (Coleoptera: Coccinellidae) from the Oriental Region. Annales Zoologici 65(2): 171–185. doi: 10.3161/00034541ANZ2015.65.2.005

[B2] Kapur AP (1948) A revision of the tribe Aspidimerini Weise (Coleoptera, Coccinellidae). Transactions of the Royal Entomological Society of London 99: 77–128. doi: 10.1111/j.1365-2311.1948.tb01233.x

[B3] Mulsant E (1850) Species des Coléoptères trimères sécuripalpes. Annales des Sciences Physiques et Naturelles, d’Agriculture et d’Industrie, Lyon (2) 2: 1–1104. doi: 10.5962/bhl.title.8953

[B4] Ślipiński A (2007) Australian ladybird beetles (Coleoptera: Coccinellidae) their biology and classification. ABRS, Canberra, 286 pp.

[B5] Ślipiński A Tomaszewska W (2010) Coccinellidae Latreille, 1802. In: Leschen RAB Beutel RG Lawrence JF (Eds) Handbook of Zoology, 2, Coleoptera. Walter de Gruyter GmbH & Co KG, Berlin, New York, 454–472.

[B6] Weise J (1900) Coccinelliden aus Ceylon gesammelt von Dr. Horn. Deutsche Entomologische Zeitschrift 44: 417–448. doi: 10.1002/mmnd.48019000237

